# New Isoform of Cardiac Myosin Light Chain Kinase and the Role of Cardiac Myosin Phosphorylation in α_1_-Adrenoceptor Mediated Inotropic Response

**DOI:** 10.1371/journal.pone.0141130

**Published:** 2015-10-29

**Authors:** Masaya Taniguchi, Ryuji Okamoto, Masaaki Ito, Itaru Goto, Satoshi Fujita, Katsuhisa Konishi, Hideo Mizutani, Kaoru Dohi, David J. Hartshorne, Takeo Itoh

**Affiliations:** 1 Department of Cardiology and Nephrology, Mie University Graduate School of Medicine, Edobashi, Tsu 514–8507, Mie, Japan; 2 Muscle Biology Group, University of Arizona, Tucson, Arizona, United States of America; 3 Department of Pharmacology, Graduate School of Medical Sciences, Nagoya City University, Nagoya 467–8601, Japan; Cinvestav-IPN, MEXICO

## Abstract

**Background & Aims:**

Cardiac myosin light chain kinase (cMLCK) plays an obligatory role in maintaining the phosphorylation levels of regulatory myosin light chain (MLC2), which is thought to be crucial for regulation of cardiac function. To test this hypothesis, the role played by ventricular MLC2 (MLC2v) phosphorylation was investigated in the phenylephrine-induced increase in twitch tension using the naturally-occurring mouse strain, C57BL/6N, in which cMLCK is down regulated.

**Methods and Results:**

By Western blot and nanoLC-MS/MS analysis, cMLCKs with molecular mass of 61-kDa (cMLCK-2) and/or 86-kDa were identified in mice heart. Among various mouse strains, C57BL/6N expressed cMLCK-2 alone and the closest relative strain C57BL/6J expressed both cMLCKs. The levels of MLC2v phosphorylation was significantly lower in C57BL/6N than in C57BL/6J. The papillary muscle twitch tension induced by electrical field stimulation was smaller in C57BL/6N than C57BL/6J. Phenylephrine had no effect on MLC2v phosphorylation in either strains but increased the twitch tension more potently in C57BL/6J than in C57BL/6N. Calyculin A increased papillary muscle MLC2v phosphorylation to a similar extent in both strains but increased the phenylephrine-induced inotropic response only in C57BL/6N. There was a significant positive correlation between the phenylephrine-induced inotropic response and the levels of MLC2v phosphorylation within ranges of 15–30%.

**Conclusions:**

We identified a new isoform of cMLCK with a molecular mass of 61kDa(cMLCK-2) in mouse heart. In the C57BL/6N strain, only cMLCK-2 was expressed and the basal MLC2v phosphorylation levels and the phenylephrine-induced inotropic response were both smaller. We suggest that a lower phenylephrine-induced inotropic response may be caused by the lower basal MLC2v phosphorylation levels in this strain.

## Introduction

The roles played by phosphorylation of the regulatory myosin light chain (MLC2) on contraction are different in cardiac and smooth muscle. In smooth muscle, MLC2 phosphorylation is Ca^2+^/calmodulin-dependent and closely related with muscle contraction [1]. In contrast, in cardiac muscle the dominant regulatory mechanism is centered on the thin-filament protein, troponin, where binding of Ca^2+^ to troponin C induces contraction. MLC2 phosphorylation plays modulatory roles [2, 3]. The spatial gradient of MLC2 phosphorylation from base to apex and from endocardium to epicardium is suggested to play a physiological role in producing cardiac torsion and maintaining normal cardiac contraction [4]. In addition, MLC2 phosphorylation may influence actin-myosin interactions independent to the actin-bound regulatory proteins [5].

The presence of cMLCK in heart was identified in 2007–2008 [6, 7]. It has so far been reported that cMLCK is an indispensable kinase for MLC2 phosphorylation in myocardium. In cultured cardiomyocytes, the knockdown of cMLCK impaired epinephrine-induced activation of sarcomere reassemble [6] and overexpression of cMLCK promoted sarcomere organization [7]. Furthermore, the lack of MLC2 phosphorylation in heart induces reduction in cardiac performance in genetically modified cMLCK knockout mice [8, 9]. Also, the results from myosin phosphatase transgenic mice and mice expressing non-phosphorylatable MLC2 in heart suggest that reduced levels of MLC2 phosphorylation induces cardiac dysfunction [10, 11]. Taken together, these findings suggest the important regulatory role of cMLCK/MLC2 phosphorylation in cardiac muscle function in vitro and in vivo. However, the roles of cMLCK/MLC2 phosphorylation in cardiac contraction under physiological conditions remain to be established.

Here, we examined whether different isoforms of cMLCK are present in hearts of various mouse strains and, if so, what kind of changes are provoked in contractile function. Using antibodies against cMLCK(41–448) and cMLCK(747–795), two reactive proteins with molecular masses of 86-kDa and 61-kDa (cMLCK-2) were identified in hearts from the C57BL/6J strain but only cMLCK-2 was identified in the C57BL/6N strain. Using these two mouse strains, the roles of MLC2v phosphorylation were studied on phenylephrine (α_1_-adrenoceptor agonist)-induced inotropic response.

## Methods

### Materials

The following chemicals were used: phenylephrine hydrochloride (Sigma Chemical Co.), guanethidine (Tokyo Kasei, Tokyo, Japan), atenolol (Sigma Chemical Co.), nifedipine (Sigma Chemical Co.), BAY K 8644 (Tocris Bioscience, Bristol, UK), ryanodine (Wako Pure Chemical, Osaka, Japan), calyculin A (Wako Pure Chemical, Osaka, Japan).

### Ethics Statement and animals

Six-week-old male mice (ICR, BALB, C3H, C57BL/6J, C57BL/6N, 20-25g) and Sprague-Dawley rats (200g) were obtained from CLEA Japan Inc. The animals were given free access to tap water and standard chow. The animals were housed under controlled temperature (21±2°C) and humidity (55±2%) in a 12:12-h light-dark cycle. Heart samples were collected after euthanasia by cervical dislocation as painlessly and quickly as possible. Animal care was performed according to the NIH guidelines (Guide for the care and use of laboratory animals), and all of the experimental protocols were approved by the Institutional Board Committee for Animal Care and Use of Mie University (Permit Number 22–45).

### Antibody production

Three antibodies against cMLCK were generated. Two polyclonal antibodies against mouse cMLCK were raised in rabbits using recombinant-GST-cMLCK(41–448aa) [cMLCK(41–448)] and recombinant-GST-cMLCK(747–795aa) [cMLCK(747–795)] as antigens. These antibodies were preabsorbed by the GST affinity column, then affinity-purified using a CNBr-activated Sepharose 4B column (GE) conjugated to the recombinant. Hybrid peptide (42-55/93-102aa, namely, WPEVLELVRA-aminohexanoic acid-LLHFQEDVTEKLQC) [cMLCK(42-55/93-102)] was synthesized and coupled with KLH. Theses amino acid sequences in cMLCK were conserved among human, bovine, dog and rodents. This rabbit anti-cMLCK antibody was prepared and affinity-purified using Epoxy-activated Sepharose 6B column (GE).

### Western blot analysis

Heart samples were extracted with modified RIPA buffer [50 mM Tris HCl pH7.5, 150 mM NaCl, 1 mM EDTA, 1% NP40, 0.25% sodium deoxycholate, 1 mM Na_3_VO_4_, 1 mM NaF, 10 mM Na_4_P_2_O_7_ and proteinase cocktail inhibitor (Complete mini, Roche)]. After centrifugation, equal volume of SDS sample buffer was added to the supernatant and heated (95°C, 5 minutes). The samples were separated by SDS-PAGE and transferred to PVDF membrane (Millipore). The three kinds of anti-cMLCK, anti-smMLCK (Sigma), anti-ZIPK (Abcam) and anti-myosin phosphatase target subunit 2 (MYPT2) [12] were used as primary antibodies. β-actin (Abcam) was used as a loading control.

### Cell culture

Cardiomyocytes were isolated from ventricles of C57BL/6J and C57BL/6N as described previously [13]. Briefly, cardiac myocytes were harvested from 1-day-old neonatal mice with the digestion of collagenase type II solution (1mg/ml, Invitrogen).The cells were cultured for 48 hours and collected for experiments.

### Measurements of MLC2 phosphorylation

In order to measure the level of MLC2 phosphorylation, cardiac samples that had been quick-frozen in liquid nitrogen was placed in a frozen slurry of TCA (10% wt/vol) in acetone plus dithiothreitol (10 mM) and allowed to thaw. The extracts were subjected to glycerol-urea PAGE followed by immunoblotting using an antibody specific for cardiac ventricular (MLC2v) antibody (BioCytex, Marseille), as described previously [10, 14]. Immunostained proteins were visualized using the ECL method.

### Northern blot analysis and RT-PCR

Total RNA was isolated from mouse heart and purified mouse cardiomyocytes by Trizol reagent (Invitrogen) according to the manufacturer’s protocol. Twenty microgram of total RNA were separated on a 1% formaldehyde agarose gel, then transferred to Hybond-N+ (GE). Northern blot probes of mouse cMLCK were generated by PCR using the following primer sets: 5’ probe forward, 5’- AGCCAGACAGGGCATCAAAT -3’, reverse, 5’- GGGACAACGTTGGAAATTTCC -3’. Membrane was hybridized to ^32^P-labeled probe in Rapid-Hyb Buffer (GE) at 65°C for 2 hours. Hybridized membrane was visualized by autoradiography. The 5’ cDNAs encoding cMLCK isoform 2 (NM_001297612.1), isoform X1(XM_011248347.1) and isoform X2(XM_006530825.2) were tried to obtain by PCR amplification using mouse cardiomyocytes random-primed cDNA as template using primer pairs 5’-AGT CGC CCC AGC CAA CCA GCC CAC C-3’(sense) and 5’-CTC CTG CCT GGG TTA GGT CAG CTC C-3’(antisense); 5’-AGA CTG TTT CTC AGT GGG AGG TGT-3’(sense) and the above same antisense; 5’-AAG CAG TCT GTG CAT GAT TTA TGC-3’ and the above same antisense respectively. The PCR conditions were 94°C for 15 sec, 58°C for 30 sec, and 68°C for 1 minute for 30 cycles using KOD pls^™^ DNA polymerase (Toyobo).

### Immunoprecipitation and nanoLC-MS/MS analysis

The heart of C57BL/6N was homogenized with modified RIPA buffer. The supernatant, after centrifugation at 13,000 X g for 10 minutes was collected and incubated with the antibody against cMLCK(41–448) for 3 hours, followed by additional incubation with protein G Sepharose (GE) for 1 hour at 4°C. The beads were washed five times with the same buffer and resuspended with SDS sample buffer. The sample was heated to 95°C for 5 minutes and subject to SDS-PAGE, followed by staining with Coomassie brilliant blue. The band for analysis was excised and underwent digestion by trypsin. The digest was acidified by trifluoroacetic acid, desalted and concentrated by ZipTipC18 Tips (Millipore). All peptides were analysed by nano-liquid chromatography-tandem mass spectrometry (nanoLC-MS/MS) using an API Applied Biosystems QSTAR XL (Framingham, MA, USA) and Bio nanoLC (KYA TECH, Tokyo, Japan). Mass spectra were decoded using the MASCOT algorithm (Matrix Science, London, UK). Homology to known or predicted proteins was assessed using the MS BLAST algorithm and annotation information in the UniProtKB/Swiss-Prot database (accessed 20 Mar 2013).

### Transthoracic echocardiography

The 10–11 week-old mice were anaesthetized with sodium pentobarbital (30 mg/kg i.p.) and examined with M-mode echocardiography in the short-axis view using a 15-MHz transducer (TOSHIBA Power Vision 6000).

### Tissue preparation and isometric tension measurement

The composition of the Krebs solution was as follows (mM): NaCl, 122; KCl, 4.7; MgCl_2_, 1.2; CaCl_2_, 2.5; NaHCO_3_, 15.5; KH_2_PO_4_, 1.2; glucose, 11.5. The solution was bubbled with 95% oxygen and 5% carbon dioxide (pH 7.3–7.4). Guanetidine (5 μM) and atenolol (1 μM) was included in Krebs solution throughout the course of experiments. The hearts were removed quickly and placed in Krebs solution. The left ventricular anterior papillary muscle was dissected using a stereoscopic microscope. The papillary muscle was mounted in a muscle chamber between a micromanipulator and force transducer. The papillary muscle was superfused with Krebs solution (at 25°C) and field stimulated (1Hz pacing frequency, voltage 1.5×threshold and 1 ms duration). After 60 minutes of equilibration, isometric twitch tension was measured. The tension per cross section was calculated and expressed as mN/mm^2^. Next, in the presence of 1 μM atenolol, phenylephrine (0.1–30 μM) was added cumulatively (concentration-response curves) or as a single bolus (10 μM) in the presence or absence of nifedipine (0.3 μM), BAY K 8644 (1 μM), ryanodine (0.3 μM) and calyculin A (10 nM) until the maximal response was obtained. These chemicals were present 20 minutes before phenylephrine stimulation. Isometric tension studies were performed using force transducers connected to Power lab/400 (AD instruments, Mountainview, CA).

### Statistics

Data are reported as the mean±standard errors of the mean (S.E.M) and were compared by Student’s *t*-test. A two-way repeated measures ANOVA was used to compare concentration-response curves to phenylephrine. A level of *P*<0.05 was considered statistically significant.

## Results

### Expression levels of cMLCK among mouse strains

**Fig 1:**
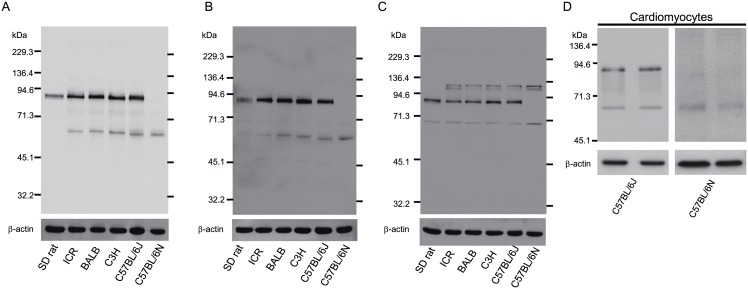
Expression of cMLCK in various mouse strains and SD rat. The expression levels of cMLCK in myocardium (A-C) and isolated cardiomyocytes (D) were shown by Western blot analysis using three kinds of cMLCK antibodies. (A) the antibody against cMLCK(41–448). (B) the antibody against cMLCK(747–795). (C) the antibody against cMLCK(42-55/93-102). β-actin was used as a loading control. (D) the antibody against cMLCK(41–448).

We found that in SD rat the antibodies against cMLCK(41–448) recognized a protein of molecular mass 86 kDa in heart (Fig 1A), but not in other tissues (brain, trachea, lung, aorta, skeletal muscle, stomach, small intestine, colon, liver, spleen, kidney and testis). Using three cMLCK antibodies, the expression of cMLCK in hearts from various mouse strains was examined. In mouse ICR, BALB, C3H and C57BL/6J strains, the two antibodies against cMLCK(41–448) and cMLCK(747–795) recognized both the 86-kDa (mainly) and a 61-kDa band (to a lesser extent) (Fig 1A and 1B). The antibody against cMLCK(42-55/93-102) recognized the 86-kDa protein, but not the 61-kDa band (Fig 1C). In contrast, in the C57BL/6N strain, the two antibodies against cMLCK(41–448) and cMLCK(747–795) reacted with the 61-kDa band, but not the 86-kDa band (Fig 1A and 1B). This is consistent with the results from isolated cardiomyocytes from heart of C57BL/6J and C57BL/6N (Fig 1D). The 86-kDa band in C57BL/6N strain was not detected by the antibody against cMLCK(42-55/93-102)(Fig 1C). The several bands (Fig 1C) of mass around 110~116-kDa and 67-kDa seem to be due to non-specific reactions, because these bands did not cross-react with the antibody against cMLCK(41–448) (Fig 1A).

### Expression of mRNA and genetic analysis

**Fig 2:**
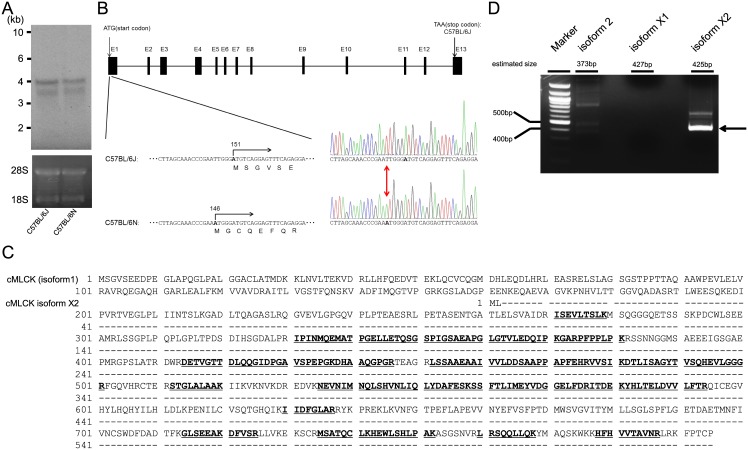
Gene expression and genome sequence analysis of Mylk3 in C57BL/6N and identification of a new isoform of cMLCK. (A) Expressions of cMLCK mRNA in C57BL/6J and C57BL/6N hearts examined by Northern blot analysis. (B) Schematic representation of Mylk3 encoding for cMLCK and identification of point mutation in C57BL/6N. The highlight indicates T146A mutation in exon1 of Mylk3. (C) Identification of cMLCK isoform. Peptides that matched sequences of cMLCK isoform 1 are shown in underlined bold. Predicted sequences of cMLCK isoform X2 is also shown. Slash bar indicates the identical sequence to cMLCK (isoform 1). (D) RT-PCR of cMLCK isoforms using cardiomyocytes. The fragment (arrow) was sequenced to confirm the existence of cMLCK isoform X2.

In Northern blot analysis in heart, two bands of 4kb and 3.5kb were detected and expression levels were not significantly different between the two strains (Fig 2A). The sequence of the cMLCK gene was then analyzed and registered, namely Mylk3, in C57BL/6N in DDBJ under accession No. AB899816. This sequence was compared with that of C57BL/6J (National Center for Biotechnology Information Gene ID: 213435). There were 41 single nucleotide polymorphisms (SNP) in C57BL/6N gene as compared with that of C57BL/6J (S1 Table). Among the SNPs detected, 40 SNPs were located in intron regions, and only one SNP located at nucleotide 146 of exon1, a T to A mutation, was detected in C57BL/6N (Fig 2B). This mutation leads to a new initiation codon, starting at 146, resulting in a frame-shift to produce a different protein of 26 amino acids (molecular weight 3.2-kDa) in C57BL/6N. This mutation might be the reason why the 86-kDa cMLCK was not expressed in C57BL/6N.

### Identification of cMLCK splicing isoform

The homogenate of C57BL/6N mouse heart was immunoprecipitated with antibody against cMLCK(41–448). The band migrating at 61-kDa in the immunoprecipitate was digested with trypsin, followed by nanoLC-MS/MS analysis. Mass spectrometry identified specific sequences as cMLCK (Fig 2C), suggesting the 61-kDa protein was originated from the mouse cMLCK products. Interestingly we could not observe any peptides deriving from N-terminus including the first methionine to 270th amino acid sequence of cMLCK isoform 1. Other significant hits represented mainly keratins and immunoglobulin (data not shown). Next we screened PubMed gene database (accessed on 2015/7/20) whether cMLCK isoforms with the preservation of carboxyl terminus of amino acids exist and found three isoforms including isoform 2, isoform X1 and isoform X2. Therefore we tried to amplify 5’ cDNAs encoding these isoforms with the use of mouse cardiomyocytes cDNA as a template. Interestingly we could recognize only isoform X2 (Fig 2D), which is the shortest isoform and composed of two amino acids (Met and Lys) substitution in the head followed by completely identical amino acid sequences of 163-795aa of cMLCK (isoform 1) with the estimated molecular mass of 69 kDa in Fig 2C. The fragment (arrow in Fig 2D) was sequenced to confirm that the PCR-amplified cDNA was identical to the original predicted sequence of isoform X2. cMLCK isoform X2 might be a possible candidate for cMLCK-2.

### MLC2v phosphorylation levels *in vivo* in C57BL/6J and C57BL/6N

**Fig 3:**
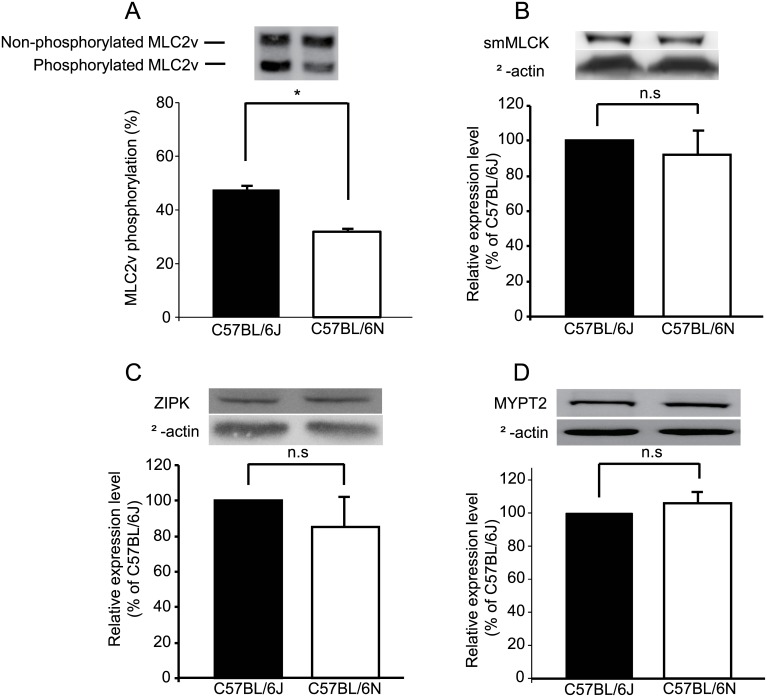
(A) Phosphorylation levels of MLC2v determined by urea-glycerol-PAGE in C57BL/6J and C57BL/6N hearts. The ratio (%) of phosphorylated MLC2v *vs*. total MLC2v was calculated. The expression of smMLCK (B), ZIPK (C) and MYPT2 (myosin phosphatase target subunit 2) (D) in C57BL/6J and C57BL/6N hearts. Upper panels show representative Western blots and the lower panels summarize the densitometrical data. The expression level in C57BL/6J was expressed as 100%. *P<0.05. *n* = 4.

The phosphorylation levels of MLC2v in the apex of the hearts were compared between C57BL/6N and C57BL/6J. The MLC2v phosphorylation in C57BL/6N (31.8±2.3%) was significantly lower than that in C57BL/6J (47.4±2.3%) (Fig 3A). The expression levels of smooth muscle MLCK (smMLCK), and zipper-interacting protein kinase (ZIPK), both of which, dominantly express in vascular smooth muscle but still present in cardiomyocytes, phosphorylate MLC2 *in vitro*, [15, 16] were not significantly different between the two strains (Fig 3B and 3C). Similarly, the expression of myosin phosphatase target subunit 2 (MYPT2) that enhances dephosphorylation of MLC2v was not significantly different between the two groups (Fig 3D).

### Comparison of morphology and cardiac function *in vivo* between C57BL/6J and C57BL/6N

Echocardiographic function in heart (left ventricular (LV) wall thickness: intraventricular septum thickness and posterior wall thickness, dimension: LV end-diastoric diameter and LV end-systolic diameter, and LV contractility: fractional shortening) was not significantly different between C57BL/6J and C57BL/6N (S2 Table).

### Effects of phenylephrine in MLC2v phosphorylation and twitch tension in isolated papillary muscle

**Fig 4:**
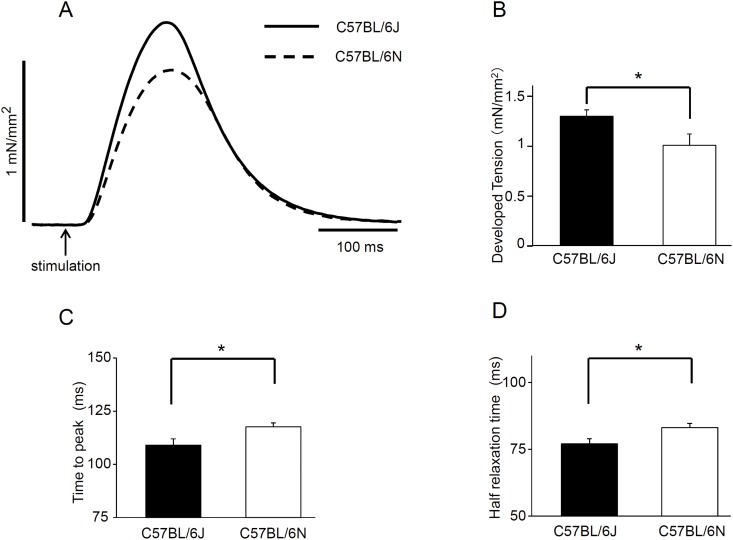
Baseline indexes for isometric tension in papillary muscles from C57BL/6J and C57BL/6N. Tension parameters were measured after 60-minute equilibration period. (A) Representative tension recordings are shown. Electrical field stimulation was made to induce twitch tension in isolated papillary muscles. The tension (B) and the time to the peak (C) or the half relaxation (D) in C57BL/6J and C57BL/6N. *P<0.05, n = 9.

Twitch tension was induced by electrical field stimulation in papillary muscle and the absolute tension was compared between C57BL/6J and C57BL/6N (Fig 4A). The twitch tension was significantly greater in C57BL/6J (1.29±0.06 mN/mm^2^) compared to C57BL/6N (1.00±0.11 mN/mm^2^, Fig 4B). The time required to peak tension was longer in C57BL/6N (118±2 msec) than in C57BL/6J (109±3 msec)(Fig 4C). Furthermore, the time required for the half relaxation was longer in C57BL/6N (83.2±1.5 msec) than in C57BL/6J (77.1±1.8 msec) (Fig 4D).

**Fig 5:**
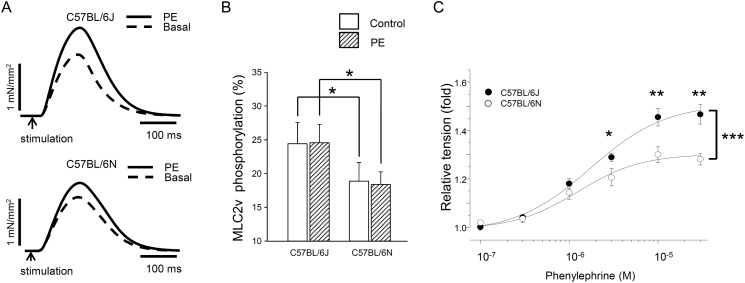
Effects of phenylephrine (PE) on twitch tension and MLC2v phosphorylation in papillary muscles from C57BL/6J and C57BL/6N. (A) Representative tension recordings. (B) The phosphorylation levels of MLC2v. The % ratio of phosphorylated MLC2v *vs*. total MLC2v was calculated (*n* = 5). (C) Concentration-dependent effects of phenylephrine in twitch tension. After basal twitch tension was recorded, phenylephrine was cumulatively applied. Atenolol was present throughout the experiments for tension measurements. *P<0.05, **P<0.01, ***P<0.001, *n* = 5–6.

Phenylephrine did not modify MLC2v phosphorylation in either C57BL/6J or C57BL/6N but it enhanced the amplitude of twitch tension in both strains (Fig 5A and 5B). Inotropic action of phenylephrine was more pronounced in C57BL/6J than in C57BL/6N (Fig 5C). No significant changes in MLC2v phosphorylation by phenylephrine were confirmed by using anti-phospho-MLC antibody (data not shown).

**Fig 6:**
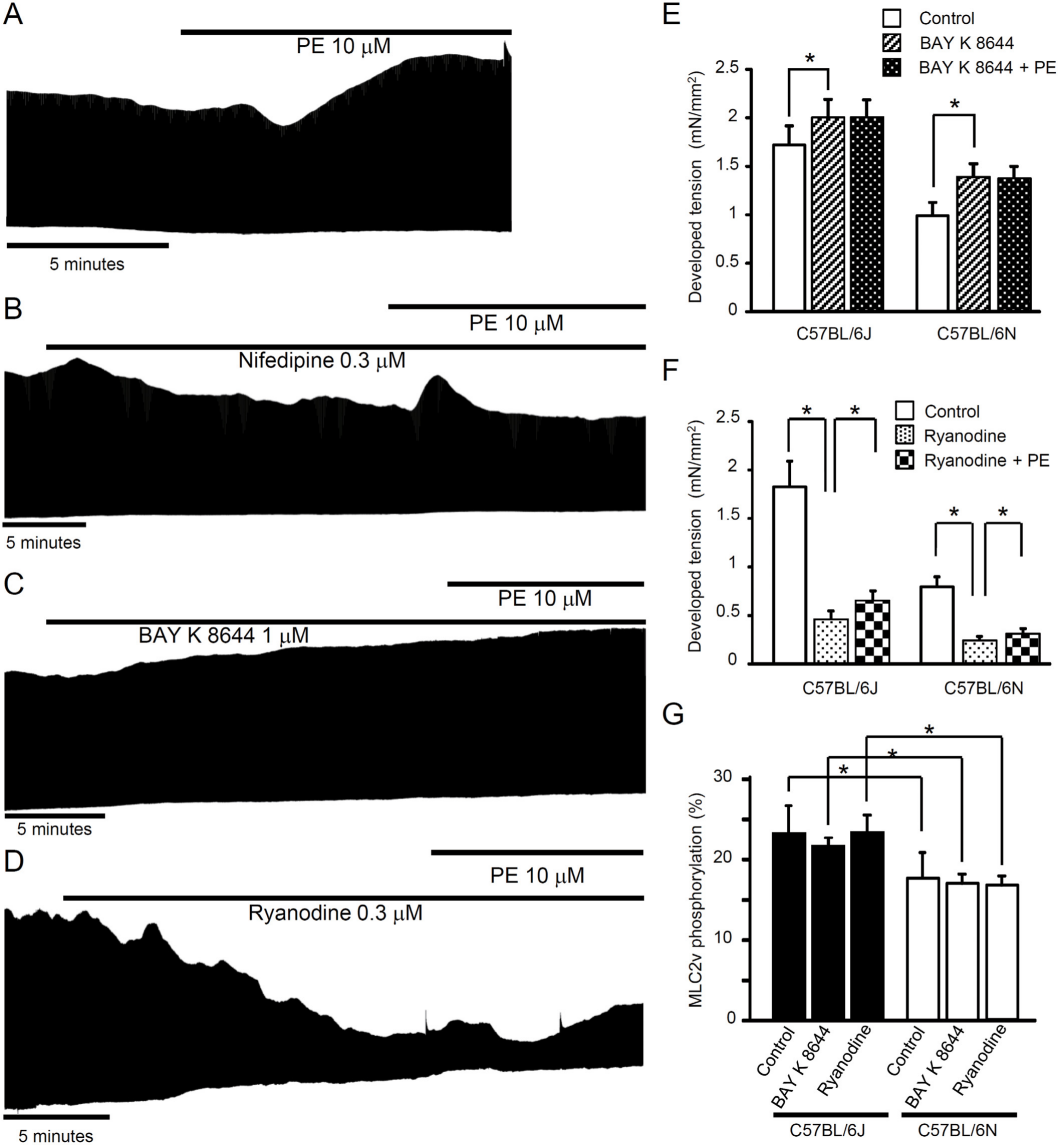
Effects of nifedipine, BAY K 8644 and ryanodine on phenylephrine-induced mechanical responses in papillary muscles. (A) The representative response to phenylephrine (10 μM) in C57BL/6J. Actions of nifedipine (0.3 μM, B), BAY K 8644 (1 μM, C) and ryanodine (0.3 μM, D) on twitch tension in the absence and presence of phenylephrine in C57BL/6J. Phenylephrine exerted positive inotropic response in the presence of ryanodine (i.e., in situations where SR-function is greatly reduced). Summary of the effects of BAY K 8644 (1 μM, E) and ryanodine (0.3 μM, F) on phenylephrine-induced response (n = 4–6, *P<0.05). (G) The levels of papillary muscle MLC2v phosphorylation in the presence and absence of BAY K 8644 or ryanodine in C57BL/6J and C57BL/6N. The % ratio of phosphorylated MLC2v *vs*. total MLC2v was calculated. BAY K 8644 and ryanodine did not modify MLC2v phosphorylation levels. The significance in the differences in the MLC2v phosphorylation levels was preserved in the presence of BAY K 8644 and ryanodine between C57BL/6J and C57BL/6N (*n* = 4–6, *P<0.05). Atenolol was present throughout the experiments for tension measurements.

Pretreatment with nifedipine (L-type Ca^2+^ channel blocker, 0.3 μM) reduced not only the twitch tension but also the phenylephrine-induced enhancement of the twitch tension in C57BL/6J (Fig 6A vs. 6B). BAY K 8644 (L-type Ca^2+^ channel activator, 1 μM) enhanced the twitch tension in both C57BL/6J and C57BL/6N, but blocked phenylephrine-induced inotropic response in both C57BL/6J and C57BL/6N (Fig 6C and 6E). Ryanodine [a blocker of Ca^2+^-induced Ca^2+^ release in sarcoplasmic reticulum (SR)] greatly reduced the basal twitch tension. In the presence of ryanodine, phenylephrine induced an inotropic action in C57BL/6J and C57BL/6N (Fig 6D and 6F).

**Fig 7:**
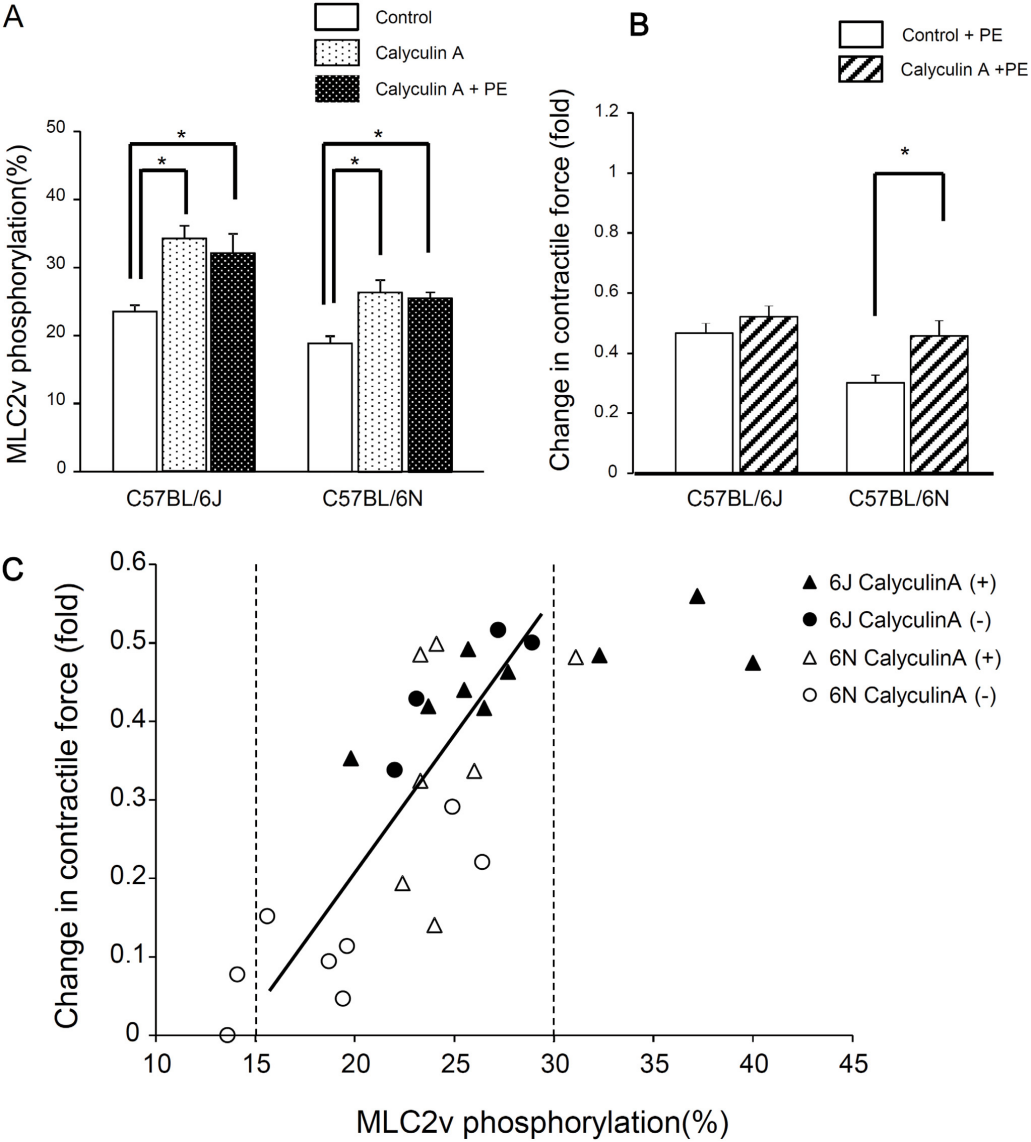
Effects of calyculin A (10 nM) on MLC2v phosphorylation and on twitch tension in the presence and absence of phenylephrine in papillary muscles from C57BL/6J and C57BL/6N. (A) MLC2v phosphorylation. Percentage ratio of phosphorylated-MLC2v *vs*. total MLC2v was calculated. *P<0.05, *n* = 4–7. (B) Twitch tension induced by phenylephrine in the presence and absence of calyculin A. *p<0.05, n = 5–6. (C) A relationship between phenylephrine-induced inotropic responses and MLC2v phosphorylation levels in the presence and absence of calyculin A. The increase in twitch tension by phenylephrine was plotted against the levels of MLC2v phosphorylation. Spearman rank correlation coefficient was calculated (by Statview software version 4.0).

The levels of MLC2v phosphorylation in papillary muscles were significantly higher in C57BL/6J than in C57BL/6N (Fig 6G). Calyculin A (an inhibitor of type 1 and 2A protein phosphatases) significantly increased the MLC2v phosphorylation in both C57BL/6J (24±1% to 34±2%) and C57BL/6N (19±1% to 26±2%) (Fig 7A). In the presence of calyculin A, inotropic responses to phenylephrine in C57BL/6N were significantly increased in proportional increase in MLC2v phosphorylation levels. In contrast, in C57BL/6J, calyculin A increased the level of MLC2v phosphorylation but did not modify the phenylephrine-induced inotropic responses (Fig 7B). The phosphorylation levels of phospholamban or troponin I in the papillary muscle were not changed after the treatment of calyculin A (S1 Fig). The relationship between the phenylephrine-induced positive inotropic response and MLC2v phosphorylation levels (within 15–30%) was positively correlated (Fig 7C).

## Discussion

We found the followings: (i) a new isoform of cMLCK (cMLCK-2) with molecular mass of 61-kDa was identified in heart from various mouse strains. (ii) C57BL/6N expressed only cMLCK-2 while other mouse strains (ICR, BALB, C3H and C57BL/6J) expressed both cMLCK-2 and 86-kDa cMLCK. (iii) The MLC2v phosphorylation levels in papillary muscle were significantly lower in C57BL/6N than in C57BL/6J. (iv) The α_1_-adrenoceptor agonist phenylephrine increased twitch tension in papillary muscle with no change in MLC2v phosphorylation in both the two mouse strains, and the response was more pronounced in C57BL/6J than in C57BL/6N. Finally, (v) the relationship was positively correlated between the phenylephrine-induced inotropic response and MLC2v phosphorylation levels (within 15–30%).

cMLCK was identified as the third MLCK in the MLCK family[6, 7]. The catalytic and the regulatory domains of cMLCK, which lie in the central and C-terminal region of the molecule, have similar sequences to skMLCK and smMLCK with identities of 58% and 44%, respectively. However, there was no significant homology between cMLCK and other MLCKs in the N-terminal sequences, where the actin-binding domain is located in smMLCK[17]. In this study two bands of molecular masses 86-kDa and 61-kDa were detected by antibodies against cMLCK(41–448) and cMLCK(747–795) in mouse ICR, BALB, C3H and C57BL/6J strains but only one band with molecular mass of 61-kDa was found in the C57BL/6N strain. The antibody against a hybrid peptide of cMLCK(42-55/93-102) cross-reacted with only 86-kDa but not the 61-kDa band. These results suggest that the 61-kDa band is a cMLCK lacking the of N-terminal sequence of cMLCK, probably due to degradation or splicing variants. In Northern blot and quantitative PCR, similar levels of cMLCK mRNAs (4kb and 3.5kb) were observed in both C57/BL/6N and its close strain C57BL/6J (data not shown). Genome sequence analysis of Mylk3 revealed 41 SNPs in C57BL/6N, one of which (T to A) was located 5 nucleotides upstream from the initiation codon in exon 1, and this mutation creates a new initiation codon at this position, resulting in a frame shift to induce a different protein with a calculated mass of 3.2 kDa. This could be the reason why the 86-kDa cMLCK is not present in C57BL/6N. With an aid of nanoLC-MS/MS analysis-Mascot search, it was found that the 61-kDa band was composed of cMLCK amino acid sequences (Fig 2C). Although the amino acid sequence of cMLCK was not obtained by the direct sequence of 61-kDa, these findings suggest that the 61-kDa species is not a degradation product of 86-kDa cMLCK. Recently, seven splicing isoforms of MYLK3 and five splicing isoforms of Mylk3 were predicted in humans and mice (Gene ID: 91807 and 213435). There are three isoforms of mouse cMLCK predicted to have conserved sequence in the carboxyl terminus of cMLCK (isoform 1). We tried to amplify and sequence the 5’ cDNA of exon 1 encoding mouse cMLCK isoform 2, isoform X1 and isoform X2 using cardiomyocytes-derived cDNA as a template and confirmed the existence of isoform X2 (Fig 2D), which is the shortest isoform among them. This isoform X2 is a possible candidate for cMLCK-2, though whether cMLCK isoform X2 is identical to 61-kDa cMLCK remains to be clarified.

The levels of MLC2v phosphorylation in papillary muscles were lower in C57BL/6N (31.8%) than in C57BL/6J (47.4%). It was found that smMLCK and ZIPK each phosphorylate MLC2v *in vitro* [16, 17]. The MLC2v phosphorylation is also regulated by myosin phosphatase. In this study it was found that the expression levels of cardiac myosin phosphatase [10] and of smMLCK, ZIPK or MYPT2 (a target subunit of cardiac myosin phosphatase) were not significantly different between C57BL/6N and C57BL/6J. Furthermore, neither the smMLCK inhibitors (ML-9 or SM1) nor the nonselective kinase inhibitor (staurosporine) or the ROCK inhibitor (Y-27632) modified papillary muscle MLC2v phosphorylation in both C57BL/6N and C57BL/6J (data not shown). It is found that SM1 inhibits both smMLCK and ZIPK [18]. In addition, ML-9 did not significantly modify the inotropic response in the papillary muscles from both C57BL/6J and C57BL/6J (data not shown). These results suggest that smMLCK, ZIPK and ROCK do not play significant roles in regulating MLC2v phosphorylation in papillary muscle for both C57BL/6N and C57BL/6J. In preliminary experiments we found that (i) the action of these inhibitors was more than a hundred fold weaker in baculovirus-expressed recombinant cMLCK than in smMLCK and (ii) ML-9 and wortmannin at 100 μM had no effect on this recombinant cMLCK (data not shown). It was also found that (iii) deletion of exon 4 or 5 in the Mylk3 gene in mice (cMLCK knockout mice) reduces MLC2v phosphorylation in heart to close to zero [9]. Taken together, these results suggest that cMLCK is the kinase responsible for phosphorylating MLC2v in papillary muscle in both C57BL/6J and C57BL/6N. More importantly, it is also suggested that the difference in papillary muscle MLC2v phosphorylation between the two mouse strains is probably due to lack of expression of the 86-kDa cMLCK in C57BL/6N.

Twitch tension induced by electrical field stimulation in papillary muscle was smaller and the time required to the peak and the half relaxation were both longer in C57BL/6N than in C57BL/6J. Phenylephrine more strongly increased the twitch tension in C57BL/6J muscle than in C57BL/6N but it had no effect on MLC2v phosphorylation level in each mouse strain. Thus, we speculate that the positive inotropic response to phenylephrine depends on the levels of basal MLC2v phosphorylation: i.e., the level of MLC2v phosphorylation under the basal conditions is higher the stronger inotropic action to phenylephrine is provoked. The results are not consistent with the previous findings that α_1_-adrenoceptor agonists increase MLC2 phosphorylation in electrically paced human atria [7, 19–21]. However the stimulation of several agonists including phenylephrine increased the level of MLC2v phosphorylation in rat cultured cardiomyocytes (S2 Fig). Our results are in contrast with the effects of ML-9 or ML-7 on the positive inotropic effect of PE, both in rat papillary muscles and human and murine atria [19–21]. These might be due to the difference in the regulatory mechanisms underlying MLC2v phosphorylation between papillary muscle and atria, cultured cardiac myocytes and papillary muscle fibers, or rat and murine, which remains to be clarified.

Nifedipine and BAY K 8644 (L-type Ca^2+^ channel blocker and activator, respectively) each blocked phenylephrine-induced inotropic response in both C57BL/6J and C57BL/6N, suggesting that the fine tuning of L-type Ca^2+^ channels may be responsible, in part, for the phenylephrine-induced inotropic response. In the presence of ryanodine (an inhibitor of Ca^2+^-induced Ca^2+^ release in SR), phenylephrine still induced an inotropic response in both strains. α1-adrenoceptor activation was reported to be able to increase Ca^2+^ influx through phosphatidylinositol turnover [22] or TRP channels [23]. From the results that both nifedipine and Bay K 8644 dispersed the enhancement of twitch by phenylephrine but its enhancement was in part conserved even in the presence of ryanodine suggested that the enhancement of twitch by phenylephrine was not due to the Ca^2+^ release from SR, but to Ca^2+^ infux at least in part through L-type Ca^2+^ channel. In a case of vascular strip, noradrenaline was reported to be able to induce arterial contraction by activating voltage-dependent Ca^2+^ channel [24].

BAY K 8644 and ryanodine did not modify papillary muscle MLC2v phosphorylation in either strains, suggesting that Ca^2+^ entry through L-type calcium channels did not influence the activity of cMLCK. The Ca^2+^-calmodulin dependency of cMLCK is controversial [5, 6]. Our preliminary data showed that baculovirus-expressed cMLCK has a basal activity in the absence of Ca^2+^/calmodulin, but its activity was enhanced by about 3.80-fold by Ca^2+^/calmodulin (data not shown), indicating that cMLCK is dependent on Ca^2+^/calmodulin. The regulation of cMLCK *in vivo* remains to be determined.

Calyculin A (an inhibitor of protein phosphatase type1 and 2A) increased MLC2v phosphorylation in cardiomyocytes [25]. It was found that calyculin A at 1 μM (but not 10 nM) increased MLC2v phosphorylation and induced an inotropic response in guinea-pig papillary muscles [26]. Here, we found that calyculin A (10 nM) increased the levels of MLC2v phosphorylation in papillary muscle to the similar magnitude in both C57BL/6J and C57BL/6N without affecting the phosphorylation levels of phospholamban and troponin I (S1 Fig and S1 Text). Thus, the absolute levels of MLC2v phosphorylation following application of calyculin A was lower in C57BL/6N than in C57BL/6J (because of the difference in the basal levels of MLC2v phosphorylation in the two strains). Calyculin A did not modify the twitch tension in either strain but it enhanced the phenylephrine-induced inotropic response in C57BL/6N more than in C57BL/6J. There was positive linear relationship between the phenylephrine-induced inotropic response and MLC2v phosphorylation levels (within a range of 15–30%, Fig 7C). These results suggest that in mouse papillary muscle the potency of inotropic response to phenylephrine is decided by the levels of MLC2v phosphorylation within the stated range. Recently the molecular mechanisms for basal MLC2v phosphorylation to induce inotropic response by α1-adrenoceptor activation was reported. MLC2v phosphorylation not only increased the proximity of cross-bridge to the thin filament but also reduced the interfilament spacing, resulting in increases in the amplitude and the Ca^2+^ sensitivity of force [27].

To understand the physiological roles of MLC2v phosphorylation in this study, the difference in the levels of MLC2v phosphorylation between the two kinds of sample should be considered. The phosphorylation levels of MLC2v in prepared papillary muscle samples was lower than those in whole heart immediately isolated from sacrificed mice (Fig 3A versus Figs 5B and 6G). This phenomenon might be due to the dephosphorylation of MLC2v during the preparation of the fiber samples and isometric tension experiments. Therefore, the tension experiments in this study does not reflect the situation in vivo, and rather shows the physiological effects of phosphorylated MLC2v in ex vivo condition in which the phosphorylation levels of MLC2v is lower than ~25%. No differences in morphology and cardiac function in vivo between the two strains support this hypothesis. Similar to our results, it is generally reported that phosphorylation levels of MLC2v in healthy heart under basal condition are ~40% [28]. By contrast, it was reported that the level of phosphorylated MLC2v were reported to be decreased to ~20% in heart failure [29, 30]. Therefore, our results obtained from the papillary muscle experiments might be linked to the pathologic condition such as heart failure.

In heart failure, the level of MLC2 phosphorylation decreases [29, 30] and as a result, cMLCK expression is enhanced [6]. Excessive chronic β_1_-adrenergic stimulation exacerbates cardiac dysfunction and decreases the expression of β_1_-adrenergic receptors, thus leading to heart failure [31, 32]. On the other hand, the expression of α_1_-adrenoceptors remain stable or rather increase in heart failure [33, 34], thus enabling maintaining the positive inotropy by α_1_-adrenoceptor agonists [35]. The levels of MLC2v phosphorylation are lower in failing heart [29, 30], and under these conditions the α_1_-adrenoceptors play an adaptive and protective role, depending on the levels of MLC2v phosphorylation. Thus, a new therapeutic strategy for heart failure could find out the way to increase and maintain MLC2v phosphorylation levels (over certain ranges).

In summary, we identified the expression of a new isoform of cMLCK with a molecular mass of 61-kDa in mouse heart. In C57BL/6J strain both 86-kDa and 61-kDa cMLCKs were expressed but only 61-kDa cMLCK was detected in C57BL/6N strain. The inotropic response to phenylephrine was closely correlated with MLC2v phosphorylation levels (within the range of 15–30%). Thus, we believe that MLC2v phosphorylation within such ranges plays an important role to induce the inotropic response by an α_1_-adrenoceptor agonist in heart under physiological conditions.

However, whether the phenomenon observed in our models, namely the MLC2v phosphorylation level-dependent inotropic response, is specific for phenylephrine or common observed in other inotropic agents including β-adrenoceptor stimulation is remained to be investigated.

## Supporting Information

S1 Fig The phoslphorylation level of phospholamban and troponin I in papillary muscles after the treatment of 10 nM Calyculin A. Phosphorylation and expression were shown by Western blot analysis.
(PDF)

S2 Fig Effects of 100 nM angiotensin II, 10 nM endothelin-1, 10 μM isoproterenol, 1 μM prostaglandin F2alpa and 50 μM phenylephrine on the phosphorylation level of MLC2v in rat neonatal cardiomyocytes. Cardiomyocytes were isolated from ventricles of 1-day-old Sprague—Dawley rat pups with the digestion of collagenase IV and trypsin. Following incubation for 24 h in serum-containing medium, the cardiomyocytes were incubated for 24 h in serum-free medium prior to stimulation with each agonist for 24 h. Phosphorylation levels were determined by glycerol-urea PAGE and Western blot analysis. n = 4–5.
(PDF)

S1 Table SNPs of Mylk3 between C57BL/6J and C57BL/6N. (DOC)

S2 Table Morphometric and echocardiographic measurements. (DOC)

S1 Text Materials. (DOC)
