# Correction: New Isoform of Cardiac Myosin Light Chain Kinase and the Role of Cardiac Myosin Phosphorylation in α1-Adrenoceptor Mediated Inotropic Response

**DOI:** 10.1371/journal.pone.0315236

**Published:** 2024-12-04

**Authors:** Masaya Taniguchi, Ryuji Okamoto, Masaaki Ito, Itaru Goto, Satoshi Fujita, Katsuhisa Konishi, Hideo Mizutani, Kaoru Dohi, David J. Hartshorne, Takeo Itoh

In Fig 2, the second amino acid should be MK in panel C. Please see the correct Fig 2 here.

**Fig 2:**
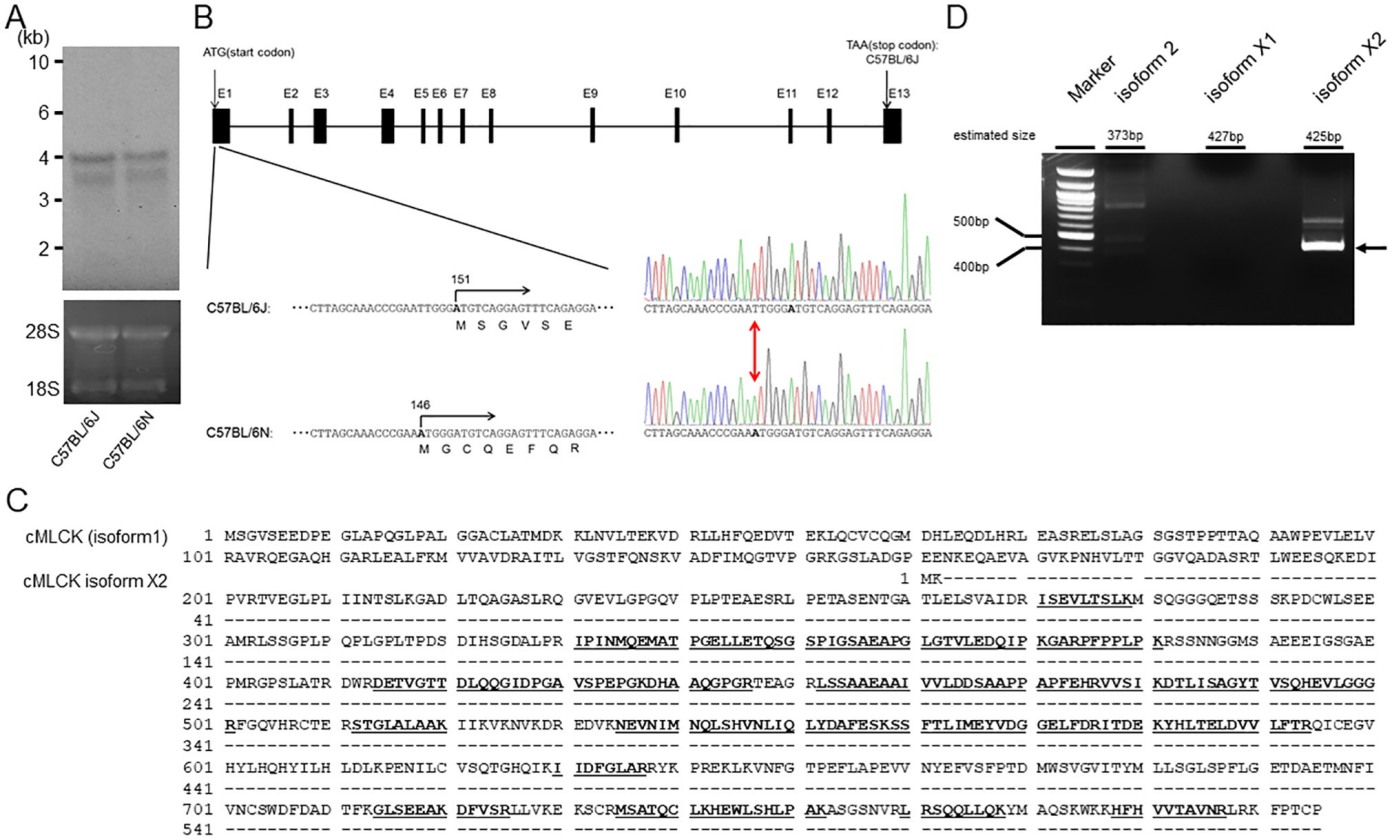
Gene expression and genome sequence analysis of Mylk3 in C57BL/6N and identification of a new isoform of cMLCK. (A) Expressions of cMLCK mRNA in C57BL/6J and C57BL/6N hearts examined by Northern blot analysis. (B) Schematic representation of Mylk3 encoding for cMLCK and identification of point mutation in C57BL/6N. The highlight indicates T146A mutation in exon1 of Mylk3. (C) Identification of cMLCK isoform. Peptides that matched sequences of cMLCK isoform 1 are shown in underlined bold. Predicted sequences of cMLCK isoform X2 is also shown. Slash bar indicates the identical sequence to cMLCK (isoform 1). (D) RT-PCR of cMLCK isoforms using cardiomyocytes. The fragment (arrow) was sequenced to confirm the existence of cMLCK isoform X2.
