# Supplementary figures and images for: New Isoform of Cardiac Myosin Light Chain Kinase and the Role of Cardiac Myosin Phosphorylation in α1-Adrenoceptor Mediated Inotropic Response
Source: PLoS One. 2015 Oct 29;10(10):e0141130. doi: 10.1371/journal.pone.0141130 (PMC4626101; doi:10.1371/journal.pone.0141130)

# Supplemental Figure S1

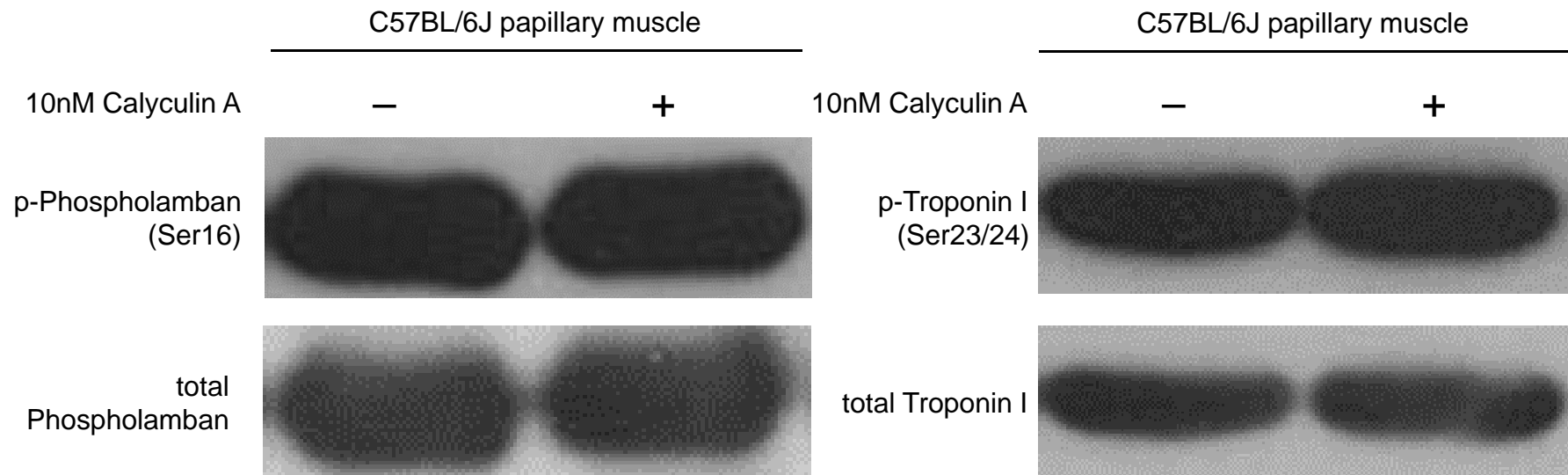

Supplement: S1 Fig — Phosphorylation and expression were shown by Western blot analysis. (PDF) [file pone.0141130.s001.pdf]

## Supplemental Figure S2

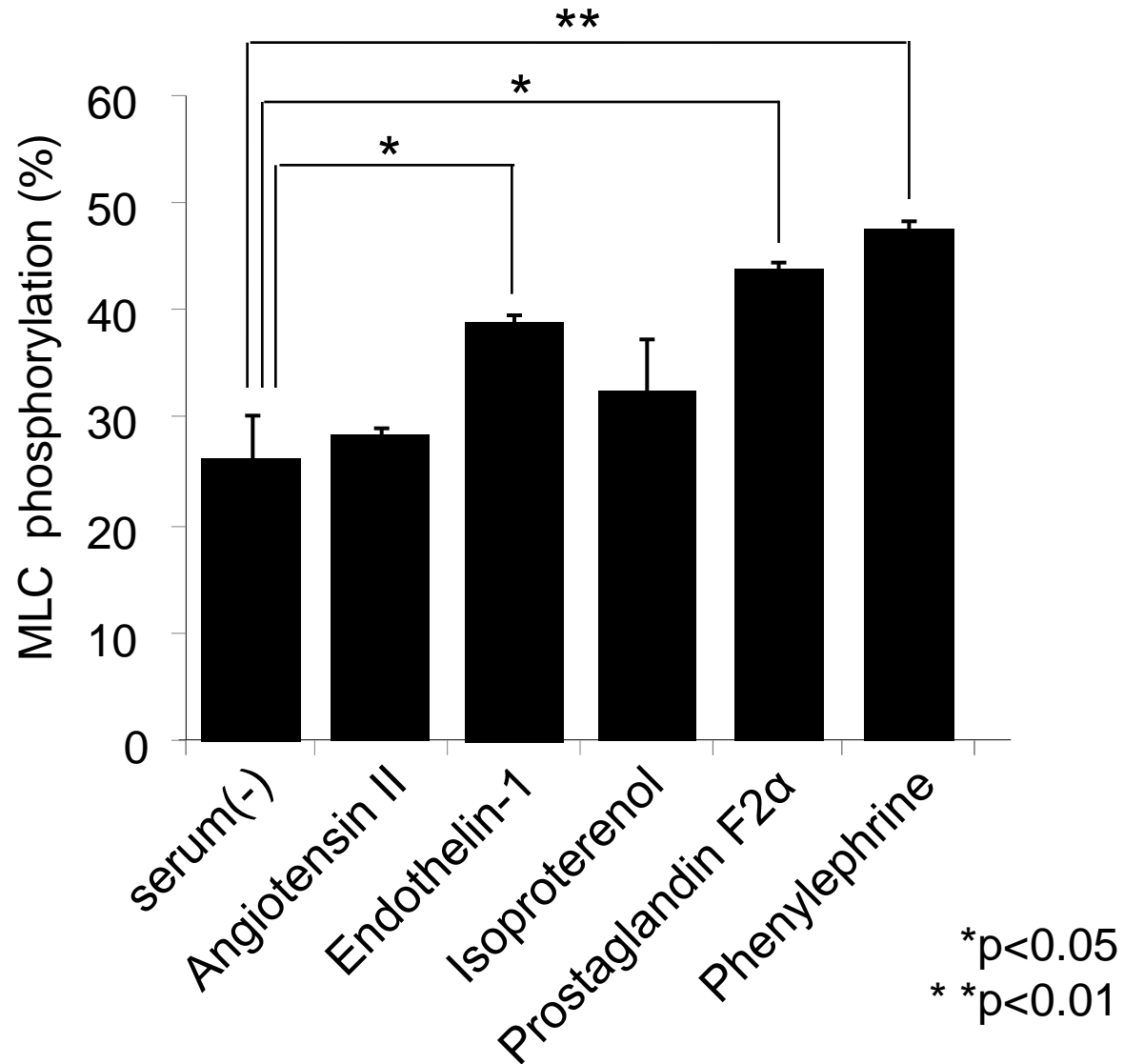

Supplement: S2 Fig — Cardiomyocytes were isolated from ventricles of 1-day-old Sprague—Dawley rat pups with the digestion of collagenase IV and trypsin. Following incubation for 24 h in serum-containing medium, the cardiomyocytes were incubated for 24 h in serum-free medium prior to stimulation with each agonist for 24 h. Phosphorylation levels were determined by glycerol-urea PAGE and Western blot analysis. n = 4–5. (PDF) [file pone.0141130.s002.pdf]
