# Supplementary material for: New Isoform of Cardiac Myosin Light Chain Kinase and the Role of Cardiac Myosin Phosphorylation in α1-Adrenoceptor Mediated Inotropic Response
Source: PLoS One. 2015 Oct 29;10(10):e0141130. doi: 10.1371/journal.pone.0141130 (PMC4626101; doi:10.1371/journal.pone.0141130)
Supplement: S1 Table — (DOC) [file pone.0141130.s003.doc]

**Supplemental Table S1.**

| Location | Genotype | | Locus |
| --- | --- | --- | --- |
|  | C57BL/6J | C57BL/6N |  |
| **87889078** | **T** | **A** | **Exon1** |
| 87873060 | T | A | Intron |
| 87872928 | A | G | Intron |
| 87872824 | T | C | Intron |
| 87872522 | T | A | Intron |
| 87872496 | T | A | Intron |
| 87872353 | C | A | Intron |
| 87872270 | A | G | Intron |
| 87872219 | A | C | Intron |
| 87872183 | A | C | Intron |
| 87872149 | T | C | Intron |
| 87871965 | T | A | Intron |
| 87870617 | A | G | Intron |
| 87870594 | T | T/C | Intron |
| 87870447 | C | T | Intron |
| 87869922 | C | A | Intron |
| 87869874 | T | T/C | Intron |
| 87869787 | T | C | Intron |
| 87869709 | T | T/C | Intron |
| 87869658 | A | A/G | Intron |
| 87869637 | G | A | Intron |
| 87869583 | G | C | Intron |
| 87869496 | G | A | Intron |
| 87869485 | C | T | Intron |
| 87869475 | T | C | Intron |
| 87869474 | G | A | Intron |
| 87869440 | G | C/G | Intron |
| 87869418 | T | C | Intron |
| 87869412 | C | T | Intron |
| 87869397 | A | T | Intron |
| 87869187 | G | A | Intron |
| 87869175 | A | G | Intron |
| 87868941 | T | C | Intron |
| 87868931 | A | T | Intron |
| 87868620 | T | A | Intron |
| 87868510 | T | C | Intron |
| 87868452 | T | G | Intron |
| 87868357 | C | A | Intron |
| 87868091 | T | G | Intron |
| 87868086 | A | G | Intron |
| 87867936 | C | T | Intron |
