# Supplementary material for: New Isoform of Cardiac Myosin Light Chain Kinase and the Role of Cardiac Myosin Phosphorylation in α1-Adrenoceptor Mediated Inotropic Response
Source: PLoS One. 2015 Oct 29;10(10):e0141130. doi: 10.1371/journal.pone.0141130 (PMC4626101; doi:10.1371/journal.pone.0141130)
Supplement: S2 Table — (DOC) [file pone.0141130.s004.doc]

**Supplemental Table S2**

|  | C57BL/6J | C57BL/6N |
| --- | --- | --- |
| Heart rate (beats/min) | 512±30 | 478±9 |
| Heart weight / body weight (mg/g) | 4.81±0.13 | 5.20±0.33 |
| IVST (mm) | 0.83±0.03 | 0.85±0.03 |
| PWT (mm) | 0.90±0.08 | 0.90±0.04 |
| LVDd (mm) | 3.33±0.15 | 3.35±0.02 |
| LVDs (mm) | 1.88±0.20 | 2.05±0.22 |
| Fractional shortening | 0.44±0.04 | 0.39±0.03 |

Values are means ± S.E.M n=4

IVST, intraventricular septum thickness; PWT, posterior wall thickness; LDVd, left ventricular end-diastolic diameter; LVDs, left ventricular end-systolic diameter.
