# Supplementary material for: New Isoform of Cardiac Myosin Light Chain Kinase and the Role of Cardiac Myosin Phosphorylation in α1-Adrenoceptor Mediated Inotropic Response
Source: PLoS One. 2015 Oct 29;10(10):e0141130. doi: 10.1371/journal.pone.0141130 (PMC4626101; doi:10.1371/journal.pone.0141130)
Supplement: S1 Text — (DOC) [file pone.0141130.s005.doc]

**Materials**

Antibodies against phospholamban (PLB; Affinity BioReagents), phospho-PLB (Ser16; Upstate), troponin I (TnI; Cell Signaling), phospho-TnI (Ser23/24; Cell Signaling) were used respectively.
